# Health Care Workers’ Legal Liability and Immunity During the COVID-19 Pandemic

**DOI:** 10.1017/dmp.2020.449

**Published:** 2020-11-19

**Authors:** Nasser Hammad Al-Azri

**Affiliations:** Emergency Department, Ibri Hospital, Ministry of Health, Muscat, Oman

**Keywords:** healthcare provider, legal liability, pandemic

## Abstract

The coronavirus disease (COVID-19) pandemic is the most unprecedented crisis facing modern health care governance in a century. Many health care activities are attracting scrutiny from ethical and legal perspectives. Therefore, health care professionals are concerned about legal ambiguity regarding legal liability and immunity in their areas of practice. Law is a key response activity that promotes a sense of safety and security among health care workers. This article describes why it is important formally to address issues of altered operations in health care practice during emergencies. Furthermore, this article provides suggestions regarding solutions to the issue of legal liability during disasters. Implementing ethical and legal clarity during a disaster response is necessary for a strong health care system at international and local levels to achieve a stable health care workforce operating for the public good within a safe and secure working environment.

During public health emergencies (PHEs), laws are designed to assign roles and responsibilities, protect vulnerable populations, and sanction critical interventions.^1^ Pandemics and disasters create new realities characterized by a health care surge (i.e., an increase in patient volume), scarce resources, transformed priorities, and the unfulfilled medical needs of communities.^2^ Since “[c]ompliance with some legal requirements becomes impossible during catastrophic disasters,” these new realities challenge medical systems, laws, and regulations, exposing their strengths and weaknesses.^3(p151)^ As a response to the recent pandemic, the *American Academy of Emergency Medicine* recently published the position statement, “Advocating for immunity from malpractice litigation during the COVID-19 pandemic.”^4^ Health care workers’ (HCWs) concerns about the legal implications of their altered operations during emergencies are both valid and warranted due to the pressure of legal ambiguity and liability, as they cross a legal minefield during their medical activities.^2,3^

The liability system aims to incentivize people and institutions to act safely and prudently by ensuring that appropriate care is provided for patients in need and compensating those who may suffer harm from inappropriate care. During a pandemic, uncertainty surrounds the potential liability risks for HCWs, including claims of negligence, breaching the standards of care, constitutional claims, criminal liability, an invasion of privacy, breaching confidentiality, the appropriate allocation of resources, licensure issues, and the right to refuse to work during a pandemic.^2,3,5,6^

HCWs seek legal protection, especially during PHEs. Some of the proposals to address liability protection include providing immunity to emergency responders, legal waivers, and Good Samaritan laws. Legal immunity, for example, involves an exemption from duty or liability.^5^ Although some laws provide civil liability protection, criminal liability immunity is commonly absent from the law. Legal waivers in existing laws represent another liability protection instrument, which legalizes certain activities under specific conditions that are otherwise considered illegal. Unfortunately, waivers can be controversial, unclear, retrospectively judged, or even ignored altogether, so they are not a dependable decision-making tool during crises.^1^ Good Samaritan laws also have severe limitations, as they are usually only applicable to those with unpaid positions or non-HCWs at the scene rather than being extended to hospital staff.^3^

Some countries have recognized and addressed the need for such legal protections. For example, the United States Public Readiness and Emergency Preparedness (PREP) Act (2005) authorizes the Department of Health during PHEs, such as pandemics, to issue a declaration providing immunity from liability for individuals and entities concerning the use of countermeasures during such emergencies.^6,7^ On March 17, 2020, the Secretary of Health and Human Services invoked the PREP Act to issue a declaration providing liability immunity applying to the current coronavirus disease (COVID-19) crisis.^8^ However, as per the Act, this liability immunity does not extend to cases of gross negligence or recklessness.

Internationally, numerous health care professionals are working in unsafe environments during the COVID-19 pandemic, which places them, their families, and their patients at risk of infection. Recognizing the vital role of HCWs’ safety, especially during PHEs, the World Health Organization (WHO) celebrated Patient Safety Day 2020 under the theme of “Health Worker Safety: A Priority for Patient Safety.”^9^ In particular, the WHO called for “speak[ing] up for health worker safety.” However, HCWs’ safety cannot be ensured without a reasonable level of legal protection for their activities during a PHE. In fact, legal ambiguity could inflict huge costs on individual HCWs, society, and the overall health care system during a PHE.

Liability issues arise in balancing the duties of HCWs and their rights, such as safe, secure working conditions and protection from liability if they elect to prioritize their safety. Furthermore, the absence of legal clarity hinders efforts to support HCW participation in emergency response.^2^ Pope and Palazzo asserted that “the law has tended to favor workers who put a patient’s needs ahead of their own.”^2^ ^(p364)^ However, there are no guarantees that this outlook will be applied in a courtroom. HCWs must be assured of their legal rights and duties to ensure the system’s strength in combatting the COVID-19 pandemic at the global, national, and institutional levels. Legal clarity becomes an ethical and legal imperative.

Unfortunately, many HCWs around the world, during PHEs and beyond, work under unsafe conditions without legal clarity of their roles and liabilities. HCWs become victims of systems that lack preparedness and capacity. Moreover, HCWs provide care during a PHE under altered crisis standards of care. Although the US Institute of Medicine referred to altered operations during PHEs as “crisis standards of care,”^10^ much ambiguity exists as to what constitutes a reasonable and prudent standard of care during the crisis itself when individual medical decision-making prevails on a case-by-case basis. Furthermore, some have argued that the standard of care is never fixed and that no single standard of care should be expected at all times.^5^ Therefore, according to this argument, there is no altered standard of care. Owing to this ambiguity, many countries still lack legally binding regulations that might offer baseline protections for their activities.

This absence of legal clarity regarding liability during a crisis hinders efforts to support HCWs’ participation in an emergency response.^2,6^ As pandemics know no borders, the ethical imperative of solidarity becomes an international mandate. A collective readiness to respond to PHEs is critical for strengthening health care systems’ preparedness and response.^11^ COVID-19 will not be the last PHE that threatens the world. Hence, it remains essential for preparedness for the next PHE to ensure HCW safety through legally binding regulations that offer the minimum legal protection for HCWs, especially at times of crises.

Law can facilitate or inhibit global efforts to combat COVID-19 and similar PHEs. In a legally binding system, the International Health Regulations (IHR) of 2005 standardized how 196 countries and the WHO may jointly address a global pandemic.^12^ Currently, the IHR document includes no provisions on safety and legal protections required for HCWs. However, this is the only international document providing an opportunity to address HCWs’ safety concerns during PHEs at the international level in a legally binding manner. Hoffmann’s suggestion may work as a basis for discussing such regulation within the IHR by considering the following statement:

They [health care workers] are acting in their capacity as [authorized] public or private entities or their agents or employees in the affected area or are volunteering under the direction of governmental authorities or [authorized] nonprofit organizations, and they are not engaged in willful misconduct, gross negligence, or criminal activity [and may be] triggered only by a declaration of a PHE.^5(p123)^

At national levels, early management of PHEs requires that public health authorities collaborate with the courts and legal systems to ensure early and appropriate education, preparation, and knowledge of procedures to deal with any situations that may arise. This can direct efforts and responses of health care systems at an earlier stage to be on the proper legal track. Furthermore, HCWs may lack an adequate, clear understanding of the existing laws. Thus, the role of education and assurance is significant as well as carefully designed, justified, and executed plans to mitigate legal ambiguity during crisis management scenarios.

Law is a pillar of the pandemic response effort that can either facilitate or inhibit global initiatives, such as combatting COVID-19 and similar PHEs. Ensuring communities’ health and safety, especially during disasters and PHEs, requires proactive measures to ensure stronger health care systems. As operating health care systems are dependent on HCWs, ensuring HCWs’ safety should be considered the cornerstone of disaster preparedness. Advocating for HCWs’ safety includes speaking up and working proactively to ensure regulations that will protect them during PHEs. As pandemics know no borders, advocacy for HCWs’ safety through legally binding regulations at the international level is more than a need – it is a legal and an ethical mandate.
